# Psychometric Properties and Measurement Invariance of the Maslach Burnout Inventory–General Survey in Colombia

**DOI:** 10.3390/ijerph18105118

**Published:** 2021-05-12

**Authors:** Diana M. Bravo, Juan C. Suárez-Falcón, Javier M. Bianchi, Miguel A. Segura-Vargas, Francisco J. Ruiz

**Affiliations:** 1 Faculty of Psychology, Fundación Universitaria Konrad Lorenz, Bogotá 110231, Colombia; dianam.bravot@konradlorenz.edu.co (D.M.B.); javierm.bianchis@konradlorenz.edu.co (J.M.B.); miguela.segurav@konradlorenz.edu.co (M.A.S.-V.); 2 Faculty of Psychology, Universidad Nacional de Educación a Distancia (UNED), 28040 Madrid, Spain; jcsuarez@psi.uned.es

**Keywords:** burnout, Maslach Burnout Inventory–General Survey, confirmatory factor analysis, measurement invariance

## Abstract

The Maslach Burnout Inventory–General Survey (MBI-GS) is a widely used scale that measures burnout in the general professions. Debate persists regarding the factor structure of the MBI-GS, and there is scarce empirical evidence about the reliability, validity, and measurement invariance of the MBI-GS in Spanish-speaking samples. Moreover, the psychometric properties of the MBI-GS have not been analyzed in Colombia. This study aimed to analyze the internal consistency, factor structure, measurement invariance, and convergent validity of the MBI-GS in a large sample of Colombian workers. The MBI-GS was administered to a total sample of 978 workers from three private companies in Bogotá (66.9% males, 32.7% females, 0.4% other). All subscales showed adequate internal consistency (alphas ranging from 0.72 to 0.86). The three-factor model demonstrated a very good fit to the data (root mean square error of approximation − RMSEA = 0.05, comparative fit index − CFI = 0.99, non-normed fit index − NNFI = 0.98, and standardized root mean square residual − SRMR = 0.06). The measurement invariance both at a metric and scalar level was supported across gender, age group, and socioeconomic status. The MBI-GS subscales showed the expected correlations with job satisfaction, work engagement, psychological distress, and psychological inflexibility. In conclusion, the Spanish version of the MBI-GS demonstrated good psychometric properties in a Colombian sample.

## 1. Introduction

In 1974, Freudenberger [1] coined the term burnout to refer to the exhaustion perceived by him and his team, who worked in a free clinic at the time. This self-perceived exhaustion not only affected their work performance but also causes detriments in other areas. Significant advances in the definition and conceptualization of burnout took place in the following decades. Burnout definitions have focused on the perception of an imbalance between the effort invested in a job versus the obtained results [2], or on the response to work-related stress and work overload once the coping strategies of the individual fail [3,4,5]. Nowadays, burnout is usually defined as a state of physical, mental, and emotional exhaustion that emerges as a result of being involved in emotionally demanding work situations [6].

The World Health Organization (WHO) [7] reported that about 40% of the world’s workers suffer from mental disorders. Burnout prevalence and incidence rates vary between studies. The prevalence in Europe is between 2.4% and 30%. Regarding studies with Spanish-speaking samples, it has been found that Spain, Argentina, and Uruguay obtained the highest prevalence rates (range = 7.9–14.9%) [8], while other Latin American countries obtained a range between 2.5% and 5.9%. Given the high prevalence rates and the disability associated with burnout, the WHO [9] announced that it will be included in 2022 as an occupational phenomenon. 

Systematic reviews have found more than 25 burnout assessment instruments (e.g., [10,11]). The Maslach Burnout Inventory (MBI [12,13]), and its later versions, is considered to be one of the most widely used instruments to measure burnout [6,14,15,16]. The original questionnaire, which later became the MBI-HSS (Human Service Survey), exclusively focused on healthcare personnel and consisted of three factors: emotional exhaustion, depersonalization, and personal accomplishment. The MBI-HSS has extensive research regarding its psychometric properties, although some debate still exists about them [17]. 

The Maslach Burnout Inventory Manual [18] introduced a version of the MBI appropriate for general workers (i.e., MBI-GS). This version involved some conceptual and theoretical restructuring. The three proposed dimensions of the MBI-GS were emotional exhaustion, cynicism, and professional efficacy [18]. Emotional exhaustion refers to a stress response to a demanding job context characterized by feelings of emotional depletion. Cynicism is a coping response to exhaustion consisting of indifferent attitudes and detachment towards the job. Lastly, the lack of professional efficacy refers to the absence of resources or skills to do the job properly [19].

The MBI-GS has been widely used and translated into multiple languages, including French, Spanish, Polish, and Dutch, among others (e.g., [4,15,16,20,21,22,23,24,25,26,27]). The psychometric properties of the MBI-GS have been studied in samples from diverse populations such as public force officials [4], engineers [23,26], teachers [16], social workers, and university students [27], among others [20,22]. These studies counted with sample sizes ranging from 114 participants to 9055 participants. Internal consistency among the studies was between acceptable and good for the full scale (*α* = 0.71–0.84), and its three dimensions (emotional exhaustion from 0.67 to 0.90, cynicism from 0.72 to 0.89; and professional efficacy from 0.69 to 0.87). 

The factor structures of the MBI and MBI-GS have been widely discussed in the empirical literature [16]. For instance, it has been suggested that burnout is a unidimensional variable [28]. In contrast, some studies have proposed a two-factor model with emotional exhaustion and cynicism collapsed in one factor (e.g., [29]). The different results regarding the factor structure of the MBI-GS might be due to the following methodological characteristics of the studies. First, the factor structure has been analyzed through exploratory factor analysis (EFA; [4,22,23,24]), confirmatory factor analysis (CFA; [14,15,25,26]), and both EFA and CFA [20,21,27]. Second, some studies have the limitations of analyzing sample sizes with low representativeness (i.e., participants from very specific populations). Third, principal component analysis with orthogonal rotations was conducted in some studies, which is incoherent with the correlated factors proposed in the original scale. Lastly, most EFA studies extracted factors with eigenvalues greater than 1 (i.e., the Kaiser rule), which usually leads to overestimating the number of dimensions (e.g., [30,31]). However, empirical evidence suggests that construct validity should be measured through the original three-factor structure besides variations in factor structure and retained items [16]. 

There is scarce empirical evidence about the reliability and validity of the MBI-GS in Spanish-speaking samples [16]. The initial studies analyzed the factor structure of the MBI-GS in small samples of participants (from 114 to 233 participants) in Spain, Cuba, and Venezuela [4,22,24,32]. These studies conducted different types of EFAs and rotation methods to explore the instrument’s factor structure, which led to different factor structures. More robust studies were conducted on Spaniard samples and in Latin American countries [16,33,34]. The first study was conducted with Spaniard samples of undergraduates and employees (total *n* = 933) [33]. The MBI-GS showed adequate internal consistency after eliminating Item 13. Moreover, the three-factor model of the MBI-GS showed an acceptable fit to the data. In the second study, the MBI-GS was administered to 978 teachers in Caribbean Spanish-speaking countries [16]. The CFAs conducted to analyze the fit of the one-, two-, and three-factor models revealed that the three-factor model obtained a better fit, especially when eliminating Item 11. The MBI-GS also showed appropriate internal consistency and convergent construct validity. A more recent psychometric study was conducted with teachers of three Latin American countries (*n* = 806): México, Perú, and Venezuela [34]. The MBI-GS demonstrated adequate internal consistency, but Item 13 was again eliminated. The three-factor model also showed a good fit to the data. Additionally, the three-factor model demonstrated measurement invariance across gender and countries. 

The last two psychometric studies were the most robust ones conducted in Spanish-speaking countries [16,34]. However, both studies have the limitation of analyzing the psychometric properties of the MBI-GS only in one profession (i.e., teachers). Overall, some limitations of the studies conducted in Spanish-speaking countries are worth noting. Firstly, there are differences across studies regarding the validity of some items, with Item 13 showing problems in some of the most robust studies [33,34]. Secondly, only one study has explored the measurement invariance of the MBI-GS [34]. Therefore, further analyses of factorial equivalence are needed. This is important because establishing measurement invariance is needed to compare the scores across different groups of participants [35]. Lastly, there are a good number of Spanish-speaking countries in which the psychometric properties of the MBI-GS have not been analyzed, such as Colombia.

In conclusion, further psychometric analyses of the MBI-GS are needed in Spanish-speaking countries. These studies should recruit large samples of different types of workers, analyze the different factor structure models through CFA, and include analyses of measurement invariance. Accordingly, this study aims to analyze the internal consistency, factor structure, measurement invariance (across gender, groupage, hierarchical level within the company, and socioeconomic status), and the convergent validity of the MBI-GS in a large sample of Colombian workers.

## 2. Materials and Methods

### 2.1. Participants

Through a non-probability convenience sampling, we recruited 978 workers from three private companies in Bogotá. The first company was a construction firm and represented 15.6% of participants. The second was a restaurant company (32.5% of participants), and the third company pertained to the automobile production sector (51.9% of participants). Most of the participants were men (66.9%, *n* = 650; females: 32.7%, *n* = 318; 0.4% of participants did not recognize themselves as male or female). The average age was 38 years old (range = 18 to 87, *SD* = 11.0), with 52.4% being older than 35. Regarding education level, 40.4% reported having completed primary or secondary studies, 24.9% were mid-level graduates (i.e., vocational training), and 19.9% were college graduates or postgraduates; 14.8% of participants did not report this information. Most of the participants (54.2%) had a low socioeconomic level, 41.0% medium, and only 4.8% had a high level. The hierarchical levels were assistance and operational (61.1%) and managerial and professional (37.5%), with 1.4% of missing data. The first hierarchical level refers to workers without a university degree who were at the lower levels of the companies. The second hierarchical level consists of workers with a university degree at medium and high levels of the company’s hierarchical structure.

### 2.2. Instruments

Maslach Burnout Inventory–General Survey (MBI-GS [18]). The MBI-GS is a 16-item, 7-point Likert-type scale (6 = *every day*; 0 = *never*) self-report instrument that assesses attitudes towards one’s work. It contains three subscales: emotional exhaustion, cynicism, and professional efficacy. High scores on emotional exhaustion and cynicism and low scores on professional efficacy are indicators of burnout. The Spanish version of the MBI-GS reported a three-factor structure and good internal consistencies across subscales in Spain (alphas between 0.85 and 0.89) [24].

Overall Job Satisfaction Scale (OJS [36]). The OJS is a scale consisting of 15 items that measure general job satisfaction through a 7-point Likert-type (7 = *very satisfied*, 1 = *very unsatisfied*). The OJS includes two factors: intrinsic satisfaction, associated with appreciation and self-fulfillment, and extrinsic satisfaction, associated with working conditions and reward. The Spanish translation [37] has shown good psychometric properties in several studies (e.g., [38]). In this study, the OJS obtained an alpha of 0.93. 

Utrecht Work Engagement Scale (UWES [39]). The UWES is a scale consisting of 15 items that measure work engagement as a construct opposed to burnout through a 7-point Likert-type scale (7 = *always true*; 1 = *never true*). The UWES comprises three factors: vigor, dedication, and absorption. The Spanish translation [40] has shown appropriate psychometric properties in several studies (e.g., [41,42]). In this study, the UWES obtained an alpha of 0.90. 

General Health Questionnaire–12 (GHQ-12 [43,44]). The GHQ-12 consists of 12 items that measure emotional distress experienced in recent weeks through a 4-point Likert-type scale. This instrument is frequently used as a screening measure for psychological disorders, with higher scores indicating higher psychological distress levels. The GHQ-12 has shown a one-factor structure and good psychometric properties in Colombian samples [43]. In this study, the GHQ-12 showed an alpha of 0.81. 

Acceptance and Action Questionnaire–II (AAQ-II [45,46]). The AAQ-II is a questionnaire consisting of 7 items and a 7-point Likert-type scale (7 = *always true*; 1 = *never true*) that evaluates psychological inflexibility (i.e., the unwillingness to experience aversive private experiences as well as the inability to be in the present moment and behave towards value-directed actions when experiencing psychological discomfort). The Spanish version showed a one-factor structure and good psychometric properties in Colombian samples [47]. In this study, the AAQ-II showed an alpha of 0.92. 

We expected negative correlations between the OJS and UWES subscales with emotional exhaustion and cynicism, and positive correlations with professional efficacy. Regarding the GHQ-12, we expected strong positive correlations between the scores in this questionnaire and emotional exhaustion and cynicism, and a negative correlation between GHQ-12 scores and professional efficacy. Lastly, according to previous research (e.g., [48]), we expected strong positive correlations between the AAQ-II scores and emotional exhaustion and cynicism. Conversely, we expected a negative correlation between the AAQ-II and professional efficacy.

### 2.3. Procedure

The companies provided permission for conducting the study with their employees during a more extensive assessment of psychosocial risk factors. All workers voluntarily agreed to participate in the study and provided informed consent. The instruments were applied during working hours. The instruments’ application was conducted in groups of 20 to 25 participants at different companies’ locations (e.g., training room, dining room, classrooms). A psychologist with a current license in occupational health in Colombia carried out the application.

Upon completion of the assessment of psychosocial risk factors, workers were invited to participate in the study. Participants who signed the informed consent were given a questionnaire package, including a sociodemographic form and the questionnaires detailed above. Participants did not receive compensation for their participation. 

### 2.4. Statistical and Psychometric Analysis

Before conducting the statistical analyses, we examined the data, searching for missing values. The data of two participants with missing values in 12 items were deleted. Afterward, we identified 90 missing data that represented 0.57% of the total number of scores. We applied the matching response pattern method of PRELIS [49] to impute these missing data. This imputation method substitutes the missing values according to a case or cases that showed a similar response pattern across the 16 items of the MBI-GS. The imputation was successful for 61 values. Nineteen participants with 29 missing values were eliminated through listwise deletion [50]. The sample size after imputation was 972 participants, with 0.39% of values imputed.

Data analysis was performed sequentially. Firstly, corrected item–total correlations were calculated, and the items with a low discrimination index (correlations below 0.30) were eliminated.

Secondly, we analyzed the dimensionality of the MBI-GS through confirmatory factor analyses (CFAs) using LISREL/WIN 8.71 (Scientific Software International Inc, Lincolnwood, IL, USA). The robust diagonally weighted least squares estimation method (Robust DWLS) was applied using polychoric correlations to perform the CFA. This estimation method is appropriate for ordinal data such as the Likert-type items of the MBI-GS. We compared the fit of three models: (a) one-factor model, (b) two-factor model with emotional exhaustion and cynicism items collapsed in the same factor, and (c) the original three-factor model. The Satorra-Bentler chi-squared test and the following goodness-of-fit indexes were calculated for all the alternative models: (a) the root mean square error of approximation (RMSEA), (b) the comparative fit index (CFI), (c) the non-normed fit index (NNFI), (d) the expected cross-validation index (ECVI), and (e) the standardized root mean square residual (SRMR). RMSEA and SRMR values of 0.08 represent a good fit, and values below 0.05 reflect a very good fit for the data [50]. For CFI and NNFI, values above 0.90 indicate well-fitting models, and values above 0.95 represent a very good fit to the data [51]. Lastly, lower ECVI values indicate a better fit to the model.

Thirdly, we analyzed construct reliability and evidence of convergent and discriminant validity of the measurement model [52,53]. Regarding construct reliability, the composite reliability coefficient (CFC) was calculated. CFC values higher than 0.70 indicate high construct reliability and adequate internal consistency. The following three criteria were analyzed regarding convergent validity of the measurement model: (a) factor loadings should be statistically significant (standardized loadings estimates should be 0.40 or higher, and ideally 0.70 or higher), (b) CFC should be higher than 0.70, and (c) the average variance extracted (AVE) should be equal or higher than 0.50 for each construct of the MBI-GS. Lastly, two criteria were considered regarding discriminant validity: (a) inter-construct correlations below 0.80 provide evidence that the discriminant validity is adequate, and (b) the square root of AVE (*√AVE*) of each factor should be greater than the inter-construct correlations with any other factor.

Fourthly, we analyzed metric and scalar invariance across gender, groupage (older than 35 vs. younger or equal than 35), hierarchical level (assistance and operational vs. managerial and professional), and socioeconomic status (low vs. medium; there were not enough participants with a high socioeconomic level) with the selected factor model. These analyses were conducted by performing further CFAs [54,55]. Metric invariance was met if the factor loadings were invariant across the sociodemographic variables, whereas scalar invariance was obtained if the item intercepts were equivalent. The multiple group baseline model, the metric invariance model, and the scalar invariance model were compared to analyze these progressively restraining models’ relative fit. The multiple-group baseline model permits the unstandardized factor loadings to be different across groups. The metric invariance model was nested within the former model and assumed equality of factor loadings across groups. Lastly, the scalar invariance model was nested within the metric invariance one and was analyzed by imposing the item intercepts and the factor loadings to be equal across groups. The RMSEA, CFI, and NNFI indexes of the previous models were compared. The more restrictive model was chosen if the following criteria were met [54,55]: (a) the difference in RMSEA (ΔRMSEA) was below 0.01 and (b) the differences in CFI (ΔCFI) and NNFI (ΔNNFI) were equivalent to or higher than −0.01. 

Fifthly, descriptive data were calculated in SPSS/WIN 25.0 (IBM Corp., Armonk, NY, USA). We computed independent samples *t*-tests to analyze if there were differences in MBI-GS scores across sociodemographic variables. Lastly, convergent construct validity was calculated using Pearson correlations between the MBI-GS subscales and the remaining self-reports. The following guidelines were used to interpret the correlations [56]: *r* between 0.10 and 0.20, small correlation; *r* between 0.21 and 0.36, medium correlation; and *r* > 0.37, strong correlation

## 3. Results

### 3.1. Descriptive Data and Psychometric Quality of the Items

Table 1 displays the corrected item-total correlations of the MBI-GS. For emotional exhaustion, they ranged from 0.66 to 0.70; for cynicism from 0.32 to 0.61; and for professional efficacy from 0.43 to 0.62. Overall, all items showed at least an adequate level of discrimination index. Cronbach’s alphas were 0.86, 0.72, and 0.79 for emotional exhaustion, cynicism, and professional efficacy, respectively.

### 3.2. Validity Evidence Based on Internal Structure

#### 3.2.1. Dimensionality

**Figure 1:**
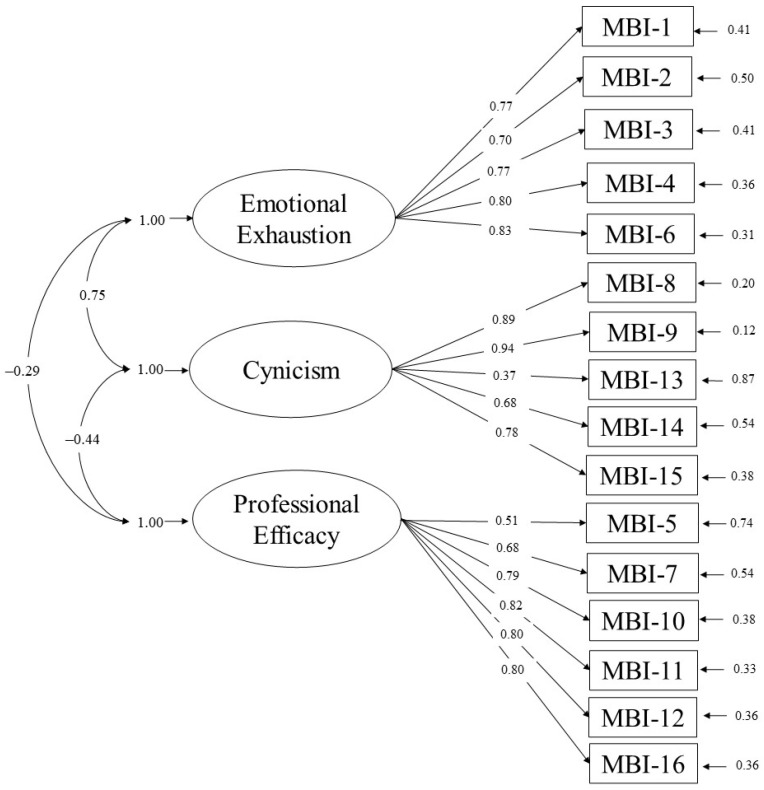
Completely standardized solution of the three-factor model.

Table 2 presents the results of the CFAs conducted with the three alternative factor models. The fit of the one-factor model was poor (RMSEA = 0.17, CFI = 0.83, NNFI = 0.81, and SRMR = 0.15), whereas the two-factor model showed an acceptable fit (RMSEA = 0.09, CFI = 0.96, NNFI = 0.95, and SRMR = 0.08). However, the fit of the three-factor model was clearly better (RMSEA = 0.05, CFI = 0.99, NNFI = 0.98, and SRMR = 0.06). The ECVI values also supported the superiority of the three-factor model (see Table 2). The results of the completely standardized solution of the three-factor model can be seen in Figure 1.

#### 3.2.2. Construct Reliability and Convergent and Discriminant Validity of the Measurement Model

The CFC values were higher than 0.70 for all factors (emotional exhaustion = 0.88, cynicism = 0.86, professional efficacy = 0.88), which indicates that the MBI-GS showed high construct reliability. 

Regarding convergent validity, all factor loadings were statistically significant, with 12 standardized loadings higher than 0.70 and three higher than 0.40. Only Item 13 showed a factor loading below the cutoff of 0.40 (i.e., lambda = 0.37). The AVE values were higher than 0.50 for the three constructs (emotional exhaustion = 0.60, cynicism = 0.58, professional efficacy = 0.55). Additionally, all CFC values were very high, supporting the convergent validity of the MBI-GS in this study. 

The discriminant validity of the MBI-GS was also supported because all inter-construct correlations were below 0.80 (emotional exhaustion and cynicism = 0.75; emotional exhaustion and professional efficacy = −0.29; cynicism and professional efficacy = −0.44). Moreover, the square roots of AVE values of each factor were higher than the inter-construct correlations of this latent variable with any other factor (√AVE of emotional exhaustion = 0.78, higher than 0.75 and −0.29; √AVE of cynicism = 0.76, higher than 0.75 and −0.44; and √AVE of professional efficacy = 0.74, higher than −0.29 and −0.44).

#### 3.2.3. Measurement Invariance

Table 3 presents the results of the measurement invariance analysis. The measurement invariance both at a metric and scalar level was supported across gender, groupage, and socioeconomic status because changes in RMSEA, CFI, and NNFI were below 0.01. Regarding the hierarchical level, all criteria were met for metric invariance. However, the criteria for scalar invariance were not fully met because the ΔRMSEA was −0.011. Therefore, we analyzed if a partial invariance scalar could be assumed by exploring the item intercepts of both groups. The modification indexes indicated that Items 9 and 13 of cynicism could be variant. Accordingly, we tested partial scalar invariance with both Item 9 and Item 13 intercepts being variants. Partial scalar invariance criteria were met for the hierarchical level groups with Items 9 and 13 with their own intercept estimations in both groups.

### 3.3. Validity Evidence Based on Relationships with Other Variables

MBI-GS subscales showed correlations in the expected directions and size with the other assessed constructs (see Table 4). Emotional exhaustion and cynicism showed medium to strong negative correlations with job satisfaction, UWES-vigor, and UWES-dedication. The correlation with UWES-absorption was small but statistically significant. As expected, these MBI-GS subscales showed strong positive correlations with psychological distress and psychological inflexibility. 

An opposite pattern of correlations was found for professional efficacy. The correlations with job satisfaction were positive and medium-size, whereas the correlations with UWES-vigor and UWES-dedication were strong. Professional efficacy correlated negatively with psychological distress and psychological inflexibility.

The MBI-GS subscales also showed the expected correlations among them. Emotional exhaustion and cynicism correlated positively and strongly. However, their correlations with professional efficacy were small but negative.

### 3.4. Descriptive Data and Differences in Scores among Sociodemographic Characteristics

Table 5 presents the descriptive data of the MBI-GS scores across gender, groupage, hierarchical level, and socioeconomic status. Statistically significant differences were found regarding gender in all MBI-GS subscales, with women showing higher scores on emotional exhaustion (*t*(571.49) = −5.41, *p* < 0.001) and cynicism (*t*(966) = −3.14, *p* = 0.002) and lower scores in professional efficacy (*t*(966) = 2.29, *p* = 0.022) than men. No statistically significant differences were found on the MBI-GS subscales according to groupage. Regarding the hierarchical level, there were no statistically significant differences in emotional exhaustion, but differences were significant for cynicism (*t*(957) = −3.39, *p* = 0.001) and professional efficacy (*t*(904.88) = 3.90, *p* < 0.001). Specifically, participants in the managerial/professional level showed less cynicism and more professional efficacy than participants in the assistance/operational level. Lastly, regarding socioeconomic status, there were no statistically significant differences in emotional exhaustion. However, participants in a low socioeconomic status showed higher scores in cynicism (*t*(924) = 2.31, *p* = 0.021) and lower scores in professional efficacy (*t*(914.70) = −2.59, *p* = 0.01) than participants in a medium status.

## 4. Discussion

The MBI-GS is one of the most widely used instruments to assess burnout in general workers. It was designed to measure three constructs: emotional exhaustion, cynicism, and professional efficacy. However, the factor structure of the MBI-GS has been discussed, which has relevant theoretical implications. There is also scarce empirical evidence of the psychometric properties of the MBI-GS in Spanish-speaking samples [16,34]. Additionally, to our best knowledge, no validation study has been conducted in Colombia. Accordingly, this study aimed to analyze the psychometric properties of the MBI-GS in a large sample of Colombian workers in three different companies (i.e., construction firm, restaurant company, and automobile production sector). 

The results of the current study showed that the MBI-GS has good psychometric properties in Colombia. The internal consistencies of the MBI-GS dimensions were adequate, and all items showed good discrimination indexes. Item 13 showed the lowest discrimination index, but contrary to other studies, it was good enough to retain the item on the scale [4,16,34]. The MBI-GS showed construct validity to the extent that the confirmatory factor analysis found that the three-factor solution obtained a very good fit to the data. The MBI-GS also demonstrated adequate construct reliability and convergent and discriminant validity of the measurement model. Overall, these findings support the original suggestion by Maslach and colleagues [18]. Thus, the current study adds evidence of the relevance of differentiating the three factors contained in the MBI-GS, particularly, differentiating Emotional Exhaustion from Cynicism. 

A relevant contribution of this study was the measurement invariance analysis included. The MBI-GS showed measurement invariance at a metric and scalar level across gender, groupage, and socioeconomic status. Metric invariance was evidenced for the hierarchical level, but only the criteria for partial scalar invariance were met because the intercepts of Items 9 and 13 were variants. 

The MBI-GS also showed convergent construct validity because it showed theoretically coherent correlations with the remaining instruments used in this study. Specifically, the subscales emotional exhaustion and cynicism correlated positively with measures of emotional distress and psychological inflexibility, and negatively with job satisfaction and work engagement. The opposite pattern of correlations was found for professional efficacy. Lastly, the findings regarding measurement invariance allowed us to compare the mean scores across groups. The highest difference found across the analyses conducted was that women showed higher levels of burnout than men. This might be due to several factors, including the work characteristics and the higher family demands usually experienced by women. Alternatively, the higher scores showed by women might be related to the characteristics of the companies analyzed. Further studies should confirm these results on a broader diversity of companies and analyze what variables could explain this gender difference in burnout. 

It is important to mention some limitations of this study. Firstly, the functioning of the MBI-GS was tested in a sample of workers from private companies. Further studies should analyze the psychometric properties of the MBI-GS in individuals from public companies and identify if similar results are found. Second, we did not obtain systematic information regarding the clinical diagnoses of the participants that would allow to compare the clinical relevance of the MBI-GS. Third, all variables analyzed in this study were measured through self-reports measures, which might have inflated the correlations obtained. Fourth, the performance of the MBI-GS was analyzed in a convenience sample, which is not representative of the working population in Colombia. Thus, the results obtained in this study should be taken with caution because they might be more a function of the conducted sampling than the actual psychometric properties of the MBI-GS in Colombia. Further studies should be conducted with additional Colombian samples to confirm the results of the current study. Lastly, the sensitivity to treatment effects of the MBI-GS was not explored. Further studies should analyze if MBI-GS scores are sensitive to the effect of psychological interventions aiming to reduce burnout. 

## 5. Conclusions

The findings of the current study are consistent with previous studies [16,18,33,34]. Specifically, the MBI-GS demonstrated adequate internal consistency, and the three-factor model showed a good fit to the data. Contrary to most of the previous psychometric studies in Spanish-speaking countries, we did not need to eliminate items of the MBI-GS. As could be expected, Item 13 from the cynicism factor showed the lowest discrimination index and standardized factor loading. However, we did not find enough evidence that would justify the elimination of this item. Indeed, the three-factor model that included Item 13 demonstrated adequate construct reliability and convergent and discriminant validity of the measurement model.

This study also adds to the empirical evidence of the measurement invariance of the three-factor model of the MBI-GS [34]. Specifically, we found scalar invariance across gender, groupage, and socioeconomic status in a large sample of different Colombian workers. This finding is important because it methodologically justifies comparing the mean scores across groups of different participants [35]. Further studies should analyze the measurement invariance of the MBI-GS across diverse samples of workers in different Spanish-speaking countries. 

In conclusion, this study showed that the MBI-GS seems to be a valid and reliable instrument in Colombian samples. Consequently, the MBI-GS appears to be an adequate measure of burnout in general workers in Colombia. 

## Figures and Tables

**Table 1:** Item description and corrected item–total correlations within each subscale.

Item Number and Description	Corrected Item–Total Correlations
1. Me siento emocionalmente agotado por mi trabajo. EE	0.68
2. Me siento acabado al final de la jornada. EE	0.66
3. Me siento fatigado al levantarme por la mañana y tener que 4. Enfrentarme a otro día de trabajo. EE	0.67
5. Trabajar todo el día realmente es estresante para mí. EE	0.66
6. Soy capaz de resolver eficazmente los problemas que surgen en mi trabajo. PE	0.43
7. Me siento quemado por mi trabajo. EE	0.70
8. Siento que estoy haciendo una contribución eficaz a la actividad de mi organización. PE	0.54
9. Desde que comencé el empleo, he ido perdiendo interés en mi trabajo. C	0.58
10. He ido perdiendo el entusiasmo en mi trabajo. C	0.61
11. En mi opinión, soy muy bueno haciendo mi trabajo. PE	0.58
12. Me siento realizado cuando llevo a cabo algo en mi trabajo. PE	0.61
13. He realizado muchas cosas que valen la pena en mi trabajo. PE	0.62
14. Sólo quiero hacer mi trabajo y que no me molesten. C	0.32
15. Me he vuelto más cínico acerca de si mi trabajo vale para algo. C	0.51
Dudo sobre el valor de mi trabajo. C	0.55
16. En mi trabajo estoy seguro de que soy eficaz haciendo las cosas. PE	0.56

Note. C = cynicism; EE = emotional exhaustion; PE = professional efficacy.

**Table 2:** Goodness-of-fit indexes of the three alternative factor models.

Goodness-of-Fit Indicators	One-Factor Model	Two-Factor Model	Three-Factor Model
RMSEA [90% CI]	0.171 [0.166, 0.176]	0.090 [0.0842, 0.0950]	0.053 [0.0469, 0.0584]
CFI	0.838	0.956	0.985
NNFI	0.813	0.949	0.982
SRMR	0.154	0.076	0.056
ECVI [90% CI]	3.218 [3.036, 3.408]	1.00 [0.905, 1.103]	0.456 [0.399, 0.520]
χ^2^ (*df*)	3060.686 (104)	904.814 (103)	372.367 (101)

Note. CFI = comparative fit index; ECVI = expected cross-validation index; NNFI = non-normed fit index; RMSEA = root mean square error of approximation; SRMR = standardized root mean square residual.

**Table 3:** Metric and scalar invariance across gender, groupage, hierarchical level, and socioeconomic status.

Model	RMSEA	ΔRMSEA	CFI	ΔCFI	NNFI	ΔNNFI
Measurement invariance across gender (men = 650, women = 318)						
MG baseline model	0.0539		0.986		0.983	
Metric invariance	0.0528	0.001	0.985	−0.001	0.984	0.001
Scalar invariance	0.0555	−0.003	0.983	−0.002	0.982	−0.002
Measurement invariance across age group (younger or equal than 35 = 454, older than 35 = 499)						
MG baseline model	0.0538		0.985		0.982	
Metric invariance	0.0532	0.001	0.984	−0.001	0.983	−0.001
Scalar invariance	0.0548	−0.002	0.983	−0.001	0.982	−0.001
Measurement invariance across hierarchical level (assistance and operational = 593, managerial and professional = 366)						
MG baseline model	0.0537		0.986		0.983	
Metric invariance	0.0526	0.001	0.986	−0.000	0.984	0.001
Scalar invariance Partial scalar invariance (9,13)	0.0635 0.0560	−0.011 0.008	0.978 0.983	−0.008 0.005	0.977 0.982	−0.007 0.005
Measurement invariance across socioeconomic status (low = 527, medium = 399)						
MG baseline model	0.0552		0.983		0.980	
Metric invariance	0.0544	0.001	0.983	0.000	0.981	0.001
Scalar invariance	0.0559	−0.002	0.981	−0.002	0.979	−0.002

**Table 4:** Pearson correlations between the MBI-GS scores and other relevant self-report measures.

Measure	Emotional Exhaustion	Cynicism	Professional Efficacy
UWES–vigor	−0.37 **	−0.30 **	0.44 **
UWES–dedication	−0.36 **	−0.40 **	0.43 **
UWES–absorption	−0.15 **	−0.12 **	0.29 **
OJS–total	−0.46 **	−0.36 **	0.28 **
OJS–intrinsic	−0.45 **	−0.33 **	0.26 **
OJS–extrinsic	−0.44 **	−0.36 **	0.28 **
GHQ–12	0.53 **	−0.43 **	−0.23 **
AAQ–II	0.42 **	−0.44 **	−0.22 **

Note. AAQ-II: Acceptance and Action Questionnaire–II; GHQ-12: General Health Questionnaire–12; MBI = Maslach Burnout Inventory; OJS = overall job satisfaction; UWES = Utrecht Work Engagement Scale; ** *p* < 0.001.

**Table 5:** Means and standard deviations across sociodemographic characteristics.

	Emotional Exhaustion	Cynicism	Professional Efficacy
Gender			
Males	6.61 (5.64)	4.89 (5.24)	30.99 (5.94)
Females	8.86 (6.30)	6.05 (5.67)	30.08 (5.59)
Groupage			
Older than 35	6.98 (5.87)	4.88 (5.40)	30.47 (6.19)
Younger or equal 35	7.73 (6.00)	5.47 (5.14)	31.01 (5.29)
Hierarchical level			
Managerial/professional	7.35 (6.02)	4.50 (5.06)	31.59 (4.88)
Assistance/operational	7.36 (5.91)	5.70 (5.51)	30.18 (6.24)
Socioeconomic status			
Low	7.63 (6.09)	5.53 (5.65)	30.37 (6.10)
Medium	7.01 (5.87)	4.71 (4.90)	31.32 (5.10)
Overall sample	7.36 (5.96)	5.27 (5.41)	30.68 (5.84)

## Data Availability

The data presented in this study are available on request from the corresponding author. The data are not publicly available for privacy reasons.
